# Evaluating the psychometric properties of the Chronic Time Pressure Inventory using Rasch analysis

**DOI:** 10.7717/peerj.15218

**Published:** 2023-04-07

**Authors:** Andrew Denovan, Neil Dagnall, Kenneth Drinkwater, Álex Escolà-Gascón

**Affiliations:** 1 People and Performance, The Manchester Metropolitan University, Manchester, United Kingdom; 2 Psychology, The Manchester Metropolitan University, Manchester, United Kingdom; 3 Applied Mathematics, Ramon Llull University, Barcelona, Spain

**Keywords:** Chronic time pressure, Chronic Time Pressure Inventory, Dimensionality, Psychometric properties, Rasch analysis

## Abstract

**Background:**

Chronic time pressure is a common source of everyday stress and anxiety. Noting this, the Chronic Time Pressure Inventory (CTPI) was designed to measure the construct within general samples. The CTPI was validated using procedures informed by classical test theory. This identified a bifactor solution, comprising a general factor encompassing two overlapping factors: Cognitive Awareness of Time Shortage and Feeling Harried. Furthermore, the CTPI demonstrated good psychometric integrity. Explicitly, internal consistency, satisfactory convergent validity with the Perceived Stress Scale, and measurement invariance. While these outcomes indicated that the CTPI was an effective measure of chronic time pressure, the scale was not subjected to analysis of item-person functioning (*i.e*., Rasch evaluation).

**Methods:**

This study accordingly examined the psychometric properties of the CTPI using Rasch analysis. A general sample of 748 (595 females, 153 males) participants completed the measure online.

**Results:**

Initial findings recommended modification of the response scale. Subsequent analyses revealed unidimensionality, adequate item/person reliability, and gender invariance. Overall, findings confirmed that the CTPI was a valid instrument for assessing perceptions of chronic time pressure within general population samples. Noting the lack of items aligning with higher ability levels, future work should develop the CTPI by adding more complex positively keyed items.

## Introduction

Perception of time shortage is a common cause of stress and anxiety within contemporary societies (Gärling et al., 2016). Acknowledging this, scholars have examined time pressure by considering several time-related constructs (*e.g*., stress, Csikszentmihalyi, 2020; deficit, Bianchi, Casper & King, 2006; and pressure, Teuchmann, Totterdell & Parker, 1999). Despite variations in terminology, scholars have often used these idioms interchangeably to refer to the subjective experience of acute time shortage. Specifically, awareness and sense of insufficient time to complete necessary tasks (Kleiner, 2014). At a personal level, this manifests as temporal overload, where individuals due to time pressure struggle or feel unable to deal with situational demands (*e.g*., deadlines and performing multiple tasks). Temporal overload comprises sense of accelerated time and constrained consideration of choices/options (Dapkus, 1985).

At a theoretical level, such perceptions reflect global time concerns (Southerton & Tomlinson, 2005), which express as feeling harried and pressed for time. In this context, harried denotes the need to undertake activities within strict timeframes, and pressed signifies deficiency of free time (Southerton & Tomlinson, 2005). Hence, harried reflects the adverse cognition of being rushed to the extent that time constraints produce anxiety and worry (Brannen, 2005). It is though important to note that perceived time pressure in some circumstances can have positive effects (*e.g*., initiating action and providing motivation). Hence, the consequences of perceived time pressure vary as a function of context, subjective experience, and duration (Rudd, 2019).

Acknowledging the absence of a universal, standardised instrument to assess time shortage, Denovan & Dagnall (2019) developed the Chronic Time Pressure Inventory (CTPI). This derived largely from the work of Szollos (2009). Conscious of the lack of conceptual clarity and coherence in the previous literature, Szollos (2009) characterized the key elements of time pressure. These originated from the observation that the subjective experience is negative (*i.e*., causes apprehension and is undesirable), and conceptualised time pressure as perceived lack of time (time shortage) and awareness of time passing swiftly (feeling rushed). Although these factors are related, they are theoretically distinct. Time shortage arises from cognitive evaluation and involves minimal emotion, whereas feeling rushed derives from subjective affective experience (*i.e*., frustration, anxiety, worry, and apprehension). This abstraction is commensurate with Szollos’s (2009) definition of time pressure as a temporal overarching designation that subsumes all terms related to time shortage as well as being rushed. Within this delineation, awareness of insufficient time (cognitive) and experience of hastened pace (affective) are clearly distinct.

Furthermore, Szollos (2009) acknowledged that time pressure was allied to stress. This aligns with the observation that acute time pressure often causes stress (Roxburgh, 2004), which can prove harmful to mental well-being (Zuzanek, 1998). This notion is consistent with individual differences research that reports relationships between stress-related time management responses (*e.g*., Type A behaviours) and personality characteristics (Frei, Racicot & Travagline, 1999). Thus, time pressure is transactional and evolves from individual interactions with the environment. This notion recognises the dynamic, subjective nature of time pressure, and the negative effect it can have on the experiencer. In this context, chronic denotes the acute nature of time pressure and that it is typically persistent, recurring, and potentially harmful (Kleiner, 2014).

Precise operationalisation of chronic time pressure is important because the construct provides an empirically informed framework for subsequent research and measurement. This includes consideration of personal (physiological) and societal (work-life balance) factors. The study of time pressure is important because it has real world applicability as demonstrated by the development of cognitive and behavioural interventions to reduce the adverse effects of chronologically induced stress. Examples include mindfulness training (to enhance work-life balance) (Kristensen, 2018), teamwork (to improve time management) (Krause et al., 2017), and downtime (to facilitate psychological detachment and physical relaxation) (Dugan & Barnes-Farrell, 2017). These illustrations highlight that chronic time pressure is an important construct generally and within specific contexts. Hence, chronic time pressure has important implications for a range of allied factors (*e.g*., well-being, Ryu, 2016; lifestyle choices, Hsu, 2015; and work-life balance, Schöneck, 2018). For instance, Gunnarsdottir et al. (2015) reported an association between parents’ subjective time pressure and increased mental health problems in children.

In the case of specific applications, researchers have investigated the effect of chronic time pressure on myriad behavioural factors such as shopping enjoyment (Kim & Kim, 2008), work engagement (Baethge et al., 2018), driving (Huang, Sun & Zhang, 2018), eating (Reichenberger et al., 2018), and street-crossing (Cœugnet, Cahour & Kraiem, 2019). Additionally, studies have evaluated the stability of chronic time pressure as a function of gender (Tyrkkö & Karlqvist, 2015), culture (Gunnarsdottir, Petzold & Povlsen, 2014), and setting (work and home) (Kleiner, 2014). Despite this wealth of research, comparisons across studies are difficult because investigators have employed a range of definitions and measures.

Illustratively, investigators have used single items (*i.e*., the General Social Survey), or small groups of questions. Typically, these are limited because they assess only general perception of time pressure or focus on specific features. For instance, the General Social Survey evaluates feeling rushed (Kleiner, 2014). Similarly, small question groups sample only restricted construct content. For example, Ackerman & Gross (2003) used a three-item measure that contained two items referring to perception of time pressure and one being harried.

Acknowledging these factors and the importance of Szollos’s (2009) conceptualisation of chronic time pressure, Denovan & Dagnall (2019) developed the Chronic Time Pressure Inventory (CTPI) using self-report methodology because this (*i.e*., questionnaires and diaries) has previously proved a valid and reliable approach to evaluating the effects of temporal pressure on individuals. Consistent with Szollos (2009), the CTPI incorporated items assessing perceived time shortage and feeling rushed. This ensured that the CTPI was theoretically grounded and allowed Denovan & Dagnall (2019) to determine the psychometric adequacies of Szollos’s (2009) proposed multidimensional model.

The CTPI was devised in accordance with psychometric conventions. Explicitly, Denovan & Dagnall (2019) devised statements that reflected the primary characteristics of time pressure. Following the removal of repetitions and unclear statements, 15 items remained. To ensure face validity (see Macaskill & Taylor, 2010), context was assessed by four experienced academics, who checked that the statements evaluated core aspects of time shortage, pressure, and feeling rushed. To permit a range of responses, participants indicated agreement *via* a five-point Likert scale, ranging from 1 (strongly disagree) to 5 (strongly agree).

Denovan & Dagnall (2019) then undertook two studies to validate the CTPI. Results supported a bifactor solution, where a general overarching factor encompassed two discrete, but overlapping temporal factors (*i.e*., Cognitive Awareness of Time Shortage and Feeling Harried). The CPTI also performed well psychometrically, demonstrating good internal consistency, satisfactory convergent validity with the Perceived Stress Scale (PSS-10; Cohen & Williamson, 1988), and measurement invariance. The inclusion of the PSS-10 was important, as it established, consistent with preceding research that time pressure positively correlated with perceived stress (Kourmousi et al., 2015). Collectively these findings supported Szollos’s (2009) conceptualization of chronic time pressure and indicated that the CTPI was a psychometrically sound instrument.

Despite these promising outcomes, additional evaluation was required because validation analysis was informed by classical test theory (CTT). The main limitation of CTT is that it is governed by the assumption that observed scores, in the absence of measurement error, represent true scores. While CTT recognises that measurement error is inevitable since no measurement instrument is perfect, some theorists criticise the underlying assumption that ensuing error is random and equally distributed across participants (Magno, 2009). Explicitly, they contend that error is systematic and a product of item difficulty. This is a key tenet of modern test theory (*i.e*., item response theory, IRT), which proposes that test scores reflect individual ability and item difficulty. Based on this premise, the Rasch modelling approach establishes expected item responses (Rasch, 1960). This applies for dichotomous (Rasch, 1960) and polytomous (Andrich, 1978) level measurement. The dichotomous model applies to ordinal data scored in two categories (typically 0 or 1), whereas the polytomous or Rasch rating scale model (RSM) was developed for data with two or more ordinal categories. For example, data collected using rating scales (*e.g*., Likert type) or other response forms with ordered categories. The polytomous model provides estimations of item difficulties, person locations, and overall set of thresholds (fixed across items).

Rasch modelling is achieved using a probabilistic form of Guttman scaling (Guttman, 1950) and fit statistics (Smith, 2000). These indicate the degree to which observed responses align with expected values. Explicitly, the probability of a respondent approving an item is presented as a logistic function, whereby the relative distance between item and respondent location on a linear scale are considered. The application of this procedure represents item responses as a combination of ability (latent trait) and difficulty. Accordingly, increased probability of a correct response reflects greater ability or smaller difficulty. Correct response probability is 0.5% when the latent trait position corresponds to item difficulty (Weller et al., 2013).

### The present study

To ensure that the CTPI factor structure identified in the original study was not contaminated by poorly performing items (*i.e*., too easy or too difficult) and differential item functioning (DIF) (Holland & Wainer, 2012), the present article used Rasch analysis. Items that are endorsed too often or too rarely are problematic because they fail to accurately separate different levels of ability. They are non-productive to measurement since respondents typically ratify or reject them. Correspondingly, these items either artificially increase or decrease overall scores (Ariffin et al., 2010; Boone, 2016). Thus, appropriate item difficulty is important to the identification of construct-based factorial structure and ensuring that relationships with other factors are accurate. Moreover, item order on the latent variable provides important information concerning construct validity (Smith, 2001).

Differential item functioning is detrimental to scale performance as it indicates that class/group designation (age, gender, ethnicity, age, education, *etc*.) rather than ability is influencing responses (Bilder & Reise, 2019). Hence, when differential item functioning is present scores are not truly indicative of ability and meaningful comparisons between respondents is not possible (de Sá Junior et al., 2019). Noting the issues associated with CTT and the advantages of the Rasch approach, the present article re-examined the psychometric properties of the CTPI.

## Materials and Methods

### Participants

A total of 748 participants took part (*M*age = 23.27, *SD* = 9.38, range = 18 to 68). The sample comprised 595 females (*M*age = 22.69, *SD* = 9.10, range = 18 to 67) and 153 males (*M*age = 25.52, *SD* = 10.12, range = 18 to 68). In terms of employment status, 209 were in full-time employment (*M*age = 30.22, *SD* = 12.73, range = 18 to 63), 242 were employed part-time (*M*age = 21.23, *SD* = 6.30, range = 18 to 59), and 297 were not currently employed (*M*age = 20.04, *SD* = 5.30, range = 18 to 68).

### Measure

#### The Chronic Time Pressure Inventory (CTPI)

The CTPI (Denovan & Dagnall, 2019) measures time pressure by focusing on the degree to which individuals perceive that they do not have adequate time to do things that they want/need to do, and the concomitant experience of feeling stressed. This is consistent with Szollos’s (2009) theoretical model. Items (*e.g*., ‘I always run out of time’) are displayed as statements with a five-point response format from 1 (strongly disagree) to 5 (strongly agree). Three items are reverse scored (2, 6, and 9). The measure contains 13 items.

### Procedure

Prospective participants were provided with a weblink, which directed them to information, which detailed the study objectives and ethical considerations. All participants who continued to the online survey provided written informed consent. The first survey section requested demographic information (age, gender, and employment status), and the second comprised the CTPI. After taking part, all participants were debriefed. To limit socially desirable responding, participants were informed that no correct responses exist. They were also told that they should work at their own pace. Ethical approval was granted by the Faculty of Health, Psychology and Social Care Ethics Committee at Manchester Metropolitan University (Project ID: 1530).

### Analysis

The psychometric properties of the CTPI were assessed using the Rasch Rating Scale Model (Andrich, 1978). Relevant items (2, 6, and 9) were reverse scored prior to analysis, consistent with scale instructions. Analyses were performed using Winsteps (Version 3.91.0; Linacre, 2015b), and progressed through several stages: scrutiny of rating scale effectiveness and reliability; dimensionality evaluation; assessment of item fit and difficulty (targeting); and analysis of differential item functioning (DIF). This is the typical approach employed when performing Rasch analyses (*e.g*., Denovan, Dagnall & Drinkwater, 2022; Royal & Elahi, 2011).

Assessment of rating scale efficacy comprises evaluation of response category usage. Adequacy was determined by monotonically increasing Rasch-Andrich thresholds and response scale Infit and Outfit statistics between 0.5 and 1.5 (Linacre, 2002). Rasch-Andrich thresholds are located at the juncture of adjoining probability curves and signify when the probability of responses being observed in one category are greater or lower than being observed in the adjoining category. Categories that are underutilised or disordered (*i.e*., suggesting that participants may be struggling to discriminate between the presented response options) can benefit from remedial action, such as collapsing response categories or treating these as missing data if a difference in valency exists.

Reliability was determined *via* indices of person separation/reliability and item separation/reliability. Separation indices provide an indication of item or participant spread on the continuum of ability and suggest the quantity of statistically different levels of item or person ability within the dataset (Bond & Fox, 2015). A minimum threshold of 1.5 exists, which indicates that the items/sample can be divided into two distinct levels (*e.g*., low, and high difficulty/ability). Reliability is analogous to Cronbach’s alpha; a minimum of 0.7 is acceptable (Fisher, 1992).

Unidimensionality of the CTPI was examined *via* Principal Components Analysis of the residuals (PCAR). PCAR has been widely utilised to assess dimensionality in Rasch models by many researchers (Han, 2022). The assumption of Rasch modelling is that a measurement model is unidimensional, and no meaningful patterns (indicative of additional factors) should exist among item residuals when controlling for the latent factor. Therefore, PCAR is a suitable and direct test of this assumption (Chou & Wang, 2010; Linacre, 2002). Several criteria were employed to determine the existence of additional factors: (i) <40% of variance accounted for by the Rasch component; (ii) >15% of variance explained by the first contrast in the residuals; and (iii) an eigenvalue of the first contrast >3 (Areepattamannil & Khine, 2018).

Infit and Outfit Mean square error (MNSQ) statistics were consulted to indicate how effectively data conformed to the Rasch model. MNSQ represents the mean value of squared residuals (*i.e*., the divergence between observed and predicted model values). Larger values signify greater misfit concerning data and model (Yan & Mok, 2012). Items with MNSQ >0.50 and <1.5 infer satisfactory fit (Linacre, 2012). A misfitting item implies that this is assessing something distinct or is inadequately defined (Smith, 1986). A quality measure should possess the capability to differentiate individuals along the continuum of ability. Thus, varying difficulties among items are required to capture this. Rasch analysis depicts targeting of item difficulty to person ability in the person-item map. Ideal targeting occurs when the mean of items corresponds with the mean of persons.

DIF exists when participants who possess equal quantities of the latent trait (chronic time pressure) respond differently to specific items. Previous research indicates that CTPI scores differ across gender (females score higher than males); this could be due to certain items attracting higher endorsement from females. The Mantel-Haenszel chi-square, its associated *p*-value, and the DIF contrast indicated the degree to which DIF occurred relative to gender (males *vs* females). Specifically, a Mantel-Haenszel *p*-value < 0.05, and a DIF contrast >0.64 represents items that evidence statistically meaningful DIF and a moderate to large effect size (Zwick, Thayer & Lewis, 1999; Linacre, 2015a). The Mantel-Haenszel chi-square tests the null hypothesis of no uniform DIF. Consideration of DIF effect size (contrast) is critical because a significant Mantel-Haenszel *p*-value in isolation is not sufficient to determine if the difference is meaningful (Boone, Staver & Yale, 2014). The presence of significant and meaningful DIF indicates the presence of bias, in the sense scale/item interpretation differs by group.

## Results

### Descriptive statistics

The mean raw CTPI score was 42.95 (*SD* = 8.17). A comparison of demographic categories (gender and employment status) revealed a statistically significant gender difference for the CTPI score, *t*(746) = 4.07, *p* < 0.001, Cohen’s *d* = 8.09, with women (*M* = 43.56, *SD* = 8.04) reporting greater chronic time pressure than men (*M* = 40.58, *SD* = 8.28). For employment status, no significant CTPI difference was found using one-way ANOVA, *F*(2,746) = 1.96, *p* = 0.142, *η*^*2*^ = 0.005; full-time employment (*M* = 43.48, *SD* = 8.46) *vs* part-time (*M* = 43.39, *SD* = 7.97) *vs* individuals not currently employed (*M* = 42.22, *SD* = 8.10).

### Rating scale and reliability

Rating scale diagnostics (Table 1) reported a satisfactory quantity of observations for each response category (*i.e*., >10) (Dimitrov, 2014; Smith et al., 2003), and average measures progressed monotonically from response category 1 (Strongly disagree) to 5 (Strongly agree). However, the Rasch-Andrich threshold values did not monotonically progress. The value of response category 3 (Neither disagree nor agree) at 0.37 was higher than category 4 (Agree) at −0.15, suggesting Rasch-Andrich threshold disorder at category 3. Therefore, category 3 responses were recoded as missing data (Linacre, 2002) because this category differed in valency *vs*. categories 2 and 4 (*i.e*., indicated neither disagreement nor agreement). Reanalysis revealed an absence of Rasch-Andrich threshold disorder and monotonic progression in accordance with response category. Accordingly, a revised response scale classification was suitable for the CTPI, consisting of ‘Strongly disagree’, ‘Disagree’, ‘Agree’, and ‘Strongly agree’. This revised response format was utilised when assessing misfit, targeting, dimensionality, and DIF.

**Table 1:** Rating scale effectiveness of the Chronic Time Pressure Inventory.

Category	Total count (%age)	Infit MNSQ	Outfit MNSQ	Average measure
1 Strongly disagree	279 (3)	1.40	1.45	−3.91
2 Disagree	2,597 (27)	0.96	0.98	−1.11
3 Neither disagree nor agree^a^	1,850 (19)			
4 Agree	3,882 (40)	0.97	0.94	1.18
5 Strongly agree	1,116 (11)	0.95	0.99	3.69

**Notes:**

Total count (%age) = total number of endorsements for a response category and the percentage of the overall total that this represents.

a Response category excluded following initial evidence of Rasch-Andrich threshold disorder.

**Figure 1:**
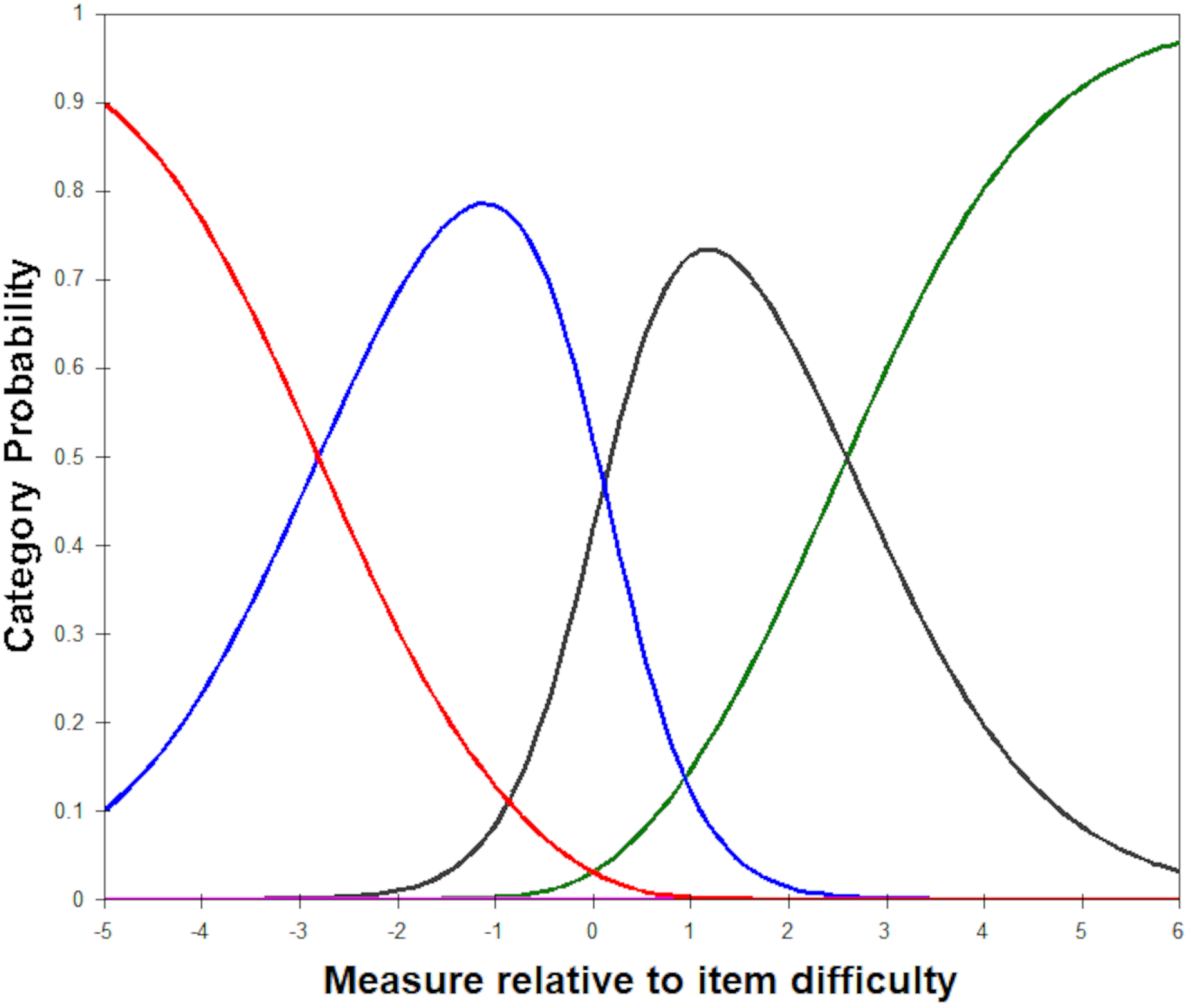
Category probability curves across all items for the Chronic Time Pressure Inventory. The central response category indicating neither disagreement nor agreement has been excluded.

Infit and outfit mean square (MNSQ) results fell between 0.5 and 1.5, indicating that the samples’ use of the four response categories was productive for measurement (DeMars, 2010; Linacre, 2015a). The category probability curves specified that all Rasch-Andrich thresholds were ordered (Fig. 1), revealing satisfactory functioning of the response scale. However, the frequencies of the different response categories in Table 1 suggests that participants were inclined to agree, rather than disagree, with items.

High measurement reliability existed (item reliability = 0.99, separation = 8.57) signifying that the sample was large enough to establish construct validity (the item hierarchy) (Linacre, 2012). Moreover, person reliability was 0.83, with a person separation of 2.17. This reflects the capacity of the CTPI to effectively distinguish between at least three levels of respondent ability.

### Dimensionality

PCAR revealed that the variance explained by the CTPI measure was satisfactory (47.1%, eigenvalue = 11.60). In addition, the percentage of variance accounted for by the first contrast was 8.7% with an eigenvalue of 2.10 (the strength of approximately two items). This finding was supportive of unidimensionality.

### Item fit and targeting

**Figure 2:**
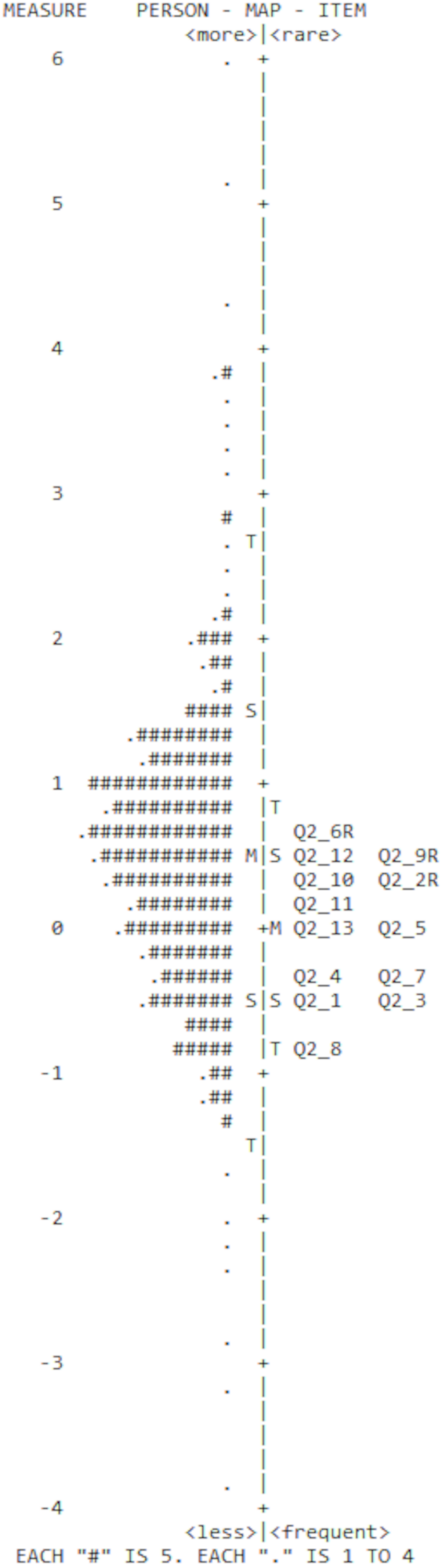
Person-item map for the Chronic Time Pressure Inventory. The participants are on the left of the dashed line and more able participants are located at the top of the map. Items are located on the right of the dashed line and more difficult items are also located at the top of the map. M, Mean persons’ ability or mean items’ difficulty; S, one standard deviation; T, two standard deviations.

No items demonstrated misfit relative to MNSQ <0.50 and >1.5 (Linacre, 2012) (Table 2), and positive and strong PTMEAs existed (>0.40). This indicated an absence of randomness within the CTPI. The person-item map (Fig. 2) represents person ability alongside item difficulty. No strong evidence of ceiling or floor effects existed, given the person mean was located at a similar level to the item mean. However, a lack of items seemed to align with higher ability levels, with only item 6 (‘I feel in control of how I spend my time’) locating above the person mean. Item 8 (‘I worry about how well I use my time’) was the easiest to endorse, whereas item 6 was the most challenging. All reverse worded items were located above the item mean, suggesting that these were more challenging to endorse on average.

**Table 2:** Item fit statistics for the Chronic Time Pressure Inventory.

Item	Difficulty	Infit MNSQ	Outfit MNSQ	PTMEA Corr.
(1) There aren’t enough hours in the day	−0.45	1.21	1.27	0.52
(2) I have enough time to do the things that I want to do	0.25	1.16	1.33	0.53
(3) I feel pressured to fit everything in	−0.46	0.90	0.88	0.61
(4) The days fly by without me ever getting everything done	−0.34	1.00	1.00	0.63
(5) I am often in a hurry	−0.04	1.01	0.99	0.59
(6) I feel in control of how I spend my time	0.59	0.84	0.87	0.67
(7) I should have more free time to do the things I enjoy	−0.26	1.25	1.31	0.52
(8) I worry about how well I use my time	−0.82	1.25	1.33	0.52
(9) I have enough time to properly prepare for things	0.58	0.84	0.87	0.63
(10) I think I won’t finish work that I set out to do	0.37	0.97	0.98	0.63
(11) I feel disappointed with how I spend my time	0.12	1.12	1.11	0.61
(12) I always run out of time	0.59	0.84	0.87	0.67
(13) I feel rushed to do the things that I have to do	0.58	0.84	0.87	0.63

**Note:**

The MNSQ acceptable limits to productive measurement were 0.5 to 1.5. Values beyond these limits are considered misfitting.

### Differential item functioning

Examination of DIF in relation to gender (Table 3) exhibited no measure issues across the scale items. Explicitly, for all items the DIF contrast (the variation in item locations) was below 0.64 logits and no significant Mantel-Haenszel values occurred. These results suggest that males and females attributed comparable meaning to the items.

**Table 3:** Differential item functioning across gender for the Chronic Time Pressure Inventory.

	Item difficulties				
Item	Men	Women	DIF contrast	Mantel-Haenszel $\chi^2$	*p*-value
1	−0.45	−0.45	0.00	0.01	0.90
2	0.29	0.25	0.04	0.74	0.38
3	−0.54	−0.46	−0.08	2.50	0.11
4	−0.47	−0.30	−0.16	0.38	0.53
5	0.07	−0.07	0.13	2.78	0.09
6	0.49	0.62	−0.14	0.00	0.97
7	−0.10	0.06	0.22	2.56	0.10
8	−0.79	−0.82	0.02	0.29	0.58
9	0.58	0.58	0.00	0.00	0.97
10	0.37	0.37	0.00	0.07	0.78
11	−0.09	.18	−0.27	2.04	0.15
12	0.58	0.42	0.16	1.01	0.31
13	0.06	0.00	0.06	0.61	0.43

## Discussion

Rasch analysis indicated that the CTPI is a psychometrically valid scale for use with general samples. Reanalysis with a modified rating scale indicated that all items were productive for measurement and functioned collectively to form a unidimensional, interval measure. Unidmensionality was verified *via* Principal Components Analysis of the residuals. Evidence of unidimensionality supports the original validation study using classical test theory (Denovan & Dagnall, 2019), which recommended using the total scale. The current findings indicate that a summary score can be used to represent chronic time pressure effectively within research.

Consideration of DIF specific to gender revealed no major measurement issues. However, women scored higher than men on the CTPI. The lack of notable gender DIF indicated that this mean difference is not necessarily a function of measurement bias, rather it is indicative of genuine sample variations (Sharp et al., 2014).

The item-person map revealed that a lack of items could be observed at the higher ability level estimates. This is an item-targeting issue and suggests that the CTPI would benefit from a greater range of item difficulty/complexity. Specifically, the inclusion of more difficult items would prove constructive to measurement. Within the CTPI, more challenging items tended to be reverse worded. This observation aligned with previous literature, which reports that negatively phrased items are more complex to comprehend (Sonderen, Sanderman & Coyne, 2013; Zhang, Noor & Savalei, 2016). Negative items typically differ in direction from most scale items. Negative wording is achieved by negating an item related to construct measurement. While this reduces habitual responding by encouraging participants to carefully consider item content, negative wording can produce confusion and/or reduce comprehensibility. Explicitly, respondents often experience difficulties processing enquires about opposite states (*i.e*., lack of chronic time pressure, feeling in control of time) (Swain, Weathers & Niedrich, 2008).

Also, the relationship between a general construct and agreeing with a negatively phrased item is not always clear. For example, endorsing item 2 (‘I have enough time to do the things that I want to do’) does not necessarily indicate the absence of chronic time pressure. High levels of agreement could be influenced by factors such as context. Hence, it is possible for an individual to endorse the item but still experience chronic time pressure. This would be the case in high-pressured work environments where individuals work specific hours. At work they would complete multiple tasks they either do not wish to undertake or have no feelings about, whereas in their free time they are able to engage fully with hobbies, pastimes, and recreational pursuits. In this instance, it is the workplace that causes the perception of chronic time pressure not life *per se*.

Despite these minor issues, overall CTPI performance was satisfactory across Rasch indices. This supports the contention of Denovan & Dagnall (2019) that the CTPI is an effective research instrument for assessing chronic time pressure. In this context, it represents an important development that will facilitate investigation in related domains such as wellbeing, stress, occupational performance, and work-life balance (Szollos, 2009). Nonetheless, future work should enhance the discriminatory power of the CTPI by adding more difficult/complex positively phrased items. Moreover, if the results concerning the functioning of the response scale are confirmed by other studies, then a modified version of the Chronic Time Pressure Inventory should be developed with a 4-point response scale (*i.e*., without the neutral central response category).

### Limitations

The study sample included less men than women (153 *vs* 595). This is a potential concern because higher CTPI existed for women. This, however, aligned with the preceding validation study (Denovan & Dagnall, 2019). Noting the gender imbalance, the study sample may have been overrepresented by higher CTPI scores. However, compared with classical test theory, Rasch analysis enables relatively sample-free standardisation of measures (Tinsley & Dawis, 1975). This indicates that gender bias is unlikely to have undermined the CTPI’s calibration. Moreover, the lower response rate of males was not unique to this study. Numerous published studies using traditional recruitment methods rather than paid participant panel providers report similar sample disparities in gender (*e.g*., Dagnall et al., 2010; Denovan et al., 2018; Lange et al., 2019).

A further limitation relates to the narrow range of demographic criteria. Only age, gender, and employment status were sampled. However, in the context of time pressure, additional variables have been identified to be significant, such as general health, occupation type, and cultural expectations. Controlling for health would preclude confounding low time pressure with individuals in poor health. Specifically, those whose low activity may chiefly determine their low time pressure. Also, research has demonstrated that time pressure can vary in accordance with occupation (Toppinen-Tanner, Kalimo & Mutanen, 2002). Furthermore, controlling for cultural expectations is important because these are value-based factors among employees, which are resultant from cultural upbringing and are outside the control of the employer (Elizur et al., 1991).

Finally, although the study sample reflected a wide age range (18 to 68), it was relatively youthful with a narrow spread (*i.e*., *M*age = 23.27, *SD* = 9.38). Sampling a greater distribution of ages and assessing these in the context of DIF would be useful for future research given that time pressure is associated with well-established changes in time perception across the life cycle (Fraisse, 1964).

## Additional Information and Declarations

### Data Availability

The following information was supplied regarding data availability: The data is available at figshare: Denovan, Andrew (2022): CTP data Rasch_1.sav. figshare. Dataset. https://doi.org/10.6084/m9.figshare.21776285.v1.
